# Range shift and introgression of the rear and leading populations in two ecologically distinct *Rubus* species

**DOI:** 10.1186/s12862-014-0209-9

**Published:** 2014-10-25

**Authors:** Makiko Mimura, Misako Mishima, Martin Lascoux, Tetsukazu Yahara

**Affiliations:** Department of Biology, Kyushu University, 6-10-1 Hakozaki, Higashi-ku, Fukuoka 812-8581 Japan; The Kyushu University Museum, 6-10-1 Hakozaki, Higashi-ku, Fukuoka 812-8581 Japan; Department of Ecology and Genetics, Uppsala University, Norbyvägen 18D, Uppsala, 75236 Sweden; Centre for Computational Systems Biology, Fudan University, 220 Handan Road, Shanghai, 200433 PRC China; Current address: Tamagawa University, 6-1-1 Tamagawa-Gakuen, Machida, Tokyo Japan

**Keywords:** Species boundaries, Introgression, Colonizer, Phylogeography, Isolation with migration, Climate change

## Abstract

**Background:**

The margins of a species’ range might be located at the margins of a species’ niche, and in such cases, can be highly vulnerable to climate changes. They, however, may also undergo significant evolutionary changes due to drastic population dynamics in response to climate changes, which may increase the chances of isolation and contact among species. Such species interactions induced by climate changes could then regulate or facilitate further responses to climatic changes. We hypothesized that climate changes lead to species contacts and subsequent genetic exchanges due to differences in population dynamics at the species boundaries. We sampled two closely related *Rubus* species, one temperate (*Rubus palmatus*) and the other subtropical (*R. grayanus*) near their joint species boundaries in southern Japan. Coalescent analysis, based on molecular data and ecological niche modelling during the Last Glacial Maximum (LGM), were used to infer past population dynamics. At the contact zones on Yakushima (Yaku Island), where the two species are parapatrically distributed, we tested hybridization along altitudinal gradients.

**Results:**

Coalescent analysis suggested that the southernmost populations of *R. palmatus* predated the LGM (~20,000 ya). Conversely, populations at the current northern limit of *R. grayanus* diverged relatively recently and likely represent young outposts of a northbound range shift. These population dynamics were partly supported by the ensemble forecasting of six different species distribution models. Both past and ongoing hybridizations were detected near and on Yakushima. Backcrosses and advanced-generation hybrids likely generated the clinal hybrid zones along altitudinal gradients on the island where the two species are currently parapatrically distributed.

**Conclusions:**

Climate oscillations during the Quaternary Period and the response of a species in range shifts likely led to repeated contacts with the gene pools of ecologically distinct relatives. Such species interactions, induced by climate changes, may bring new genetic material to the marginal populations where species tend to experience more extreme climatic conditions at the margins of the species distribution.

**Electronic supplementary material:**

The online version of this article (doi:10.1186/s12862-014-0209-9) contains supplementary material, which is available to authorized users.

## Background

Some of the most dramatic evolutionary changes within a species’ range can occur at its distributional margins where individuals tend to be exposed to extreme environments within the species’ range. Crucial processes such as hybridization, population fragmentation and increased genetic drift may occur more often at the margins than in the core of a species’ range [1–3]. Changes in climate that force the species’ range to shift might even increase these dynamics through species interactions.

Past climate changes have had significant effects on the distribution of species, and the locations of species margins have fluctuated through time [4]. At the end of the last glacial period, species ranges generally shifted towards higher latitudes, so that today, one might be able to distinguish a leading edge and a rear edge. Woody species recursively tracked the changes in climate during the latest deglaciation [5]. Hampe and Petit [6] argue that the rear edge populations have sometimes persisted over long periods of time and have, therefore, been more stable than other populations from the range. A meta-analysis revealed that lower latitudinal populations of a species tended to show a higher degree of divergence between populations, even after controlling for geographic distance [7]. This implies that these populations at the current rear margins often persist in isolation during a species’ range shift. In contrast, populations at the current leading edge tend to expand and may not be isolated for long periods. Because different species/populations react in different ways in response to climate changes and have ranges covering different latitudes, the rear edge of a species may, at some point, be in contact with the leading edge of another. Consequently, such environmental change may result in sympatry and be due to the interaction of once geographically isolated species at their boundaries.

A relatively large proportion of extant biodiversity diverged recently (less than 5 million years old), and these relatively young species may be particularly susceptible to hybridization due to the absence of postzygotic isolation [8]. Most hybrids have lower fitness than parental species in the parental niches [9], but they could be also equivalent to, or have higher fitness than, parental species depending on hybrid genotypes and environments [10]. Nonetheless, hybridization with viable hybrid offspring occurs in roughly 10% and 25% of animal and plant species, respectively [11], and subsequent hybridizations between hybrids and parental species, which result in gene transfer into recipient species (i.e., introgression), can sometimes lead to adaptive evolution [12–15]. A recent study suggests that contemporary climate changes could force a species’ range to expand and induce hybridization [16]. Climate oscillations must have induced repeated range shifts [4], and may have increased the chance of hybridization and introgression between the species at their boundaries. Since hybridization can be a major evolutionary driver [17,18], contacts between species of recent evolutionary origins and subsequent gene flow may be a common feature of biodiversity dynamics at range margins. This species interaction (i.e. hybridization) newly induced by climate changes as a result of the response of the species may ultimately contribute to increased genetic variation and provide genetic resources when the species undergoes natural selection in changing environments.

Yakushima (Yaku Island) offers a unique environment for testing differences in impact of past and contemporary climate change on range distribution and its evolutionary consequences at the northern and southern limits of species range. Due to its latitude (N30° 20’) and range of altitudes (~1936 m), the island harbors vegetation types ranging from subtropical to alpine, and serves as the southern limit of more than 200 temperate plant species in Japan (UNESCO; http://whc.unesco.org/en/list/662), and as the northernmost limit for some subtropical-tropical species that are distributed from mainland China to southern Japan through a string of islands.

*Rubus palmatus* Thunb., and *Rubus grayanus* Maximowicz are wild raspberry species distributed in Asia, including Yakushima Island. *R. palmatus* is an endemic temperate species in Japan and is divided into three varieties: var. *palmatus* in western Japan, var. *coptophyllus* in north-eastern Japan, and var. *yakumontanus* on Yakushima Island. *R. grayanus* is distributed in subtropical climates including southeast China and Japan. Both species are diploid species belonging to the genus *Rubus,* subgenus *Ideaobatus*. A major ecological difference between these species is in leaf seasonality (deciduous and evergreen). The species co-exist only on Yakushima Island, where the southernmost population of *R. palmatus* is present at high altitude and the northernmost population of *R. grayanus* is present at low altitude. Yakushima Island was connected with the southern Kyushu Island during the Last Glacial Maximum (LGM), and putatively served as refugia for temperate forests [19]. This implies that some temperate species, including *R. palmatus,* persisted as marginal populations over the glaciation period on and around the island. If postglacial climate change led to parapatry of these *Rubus* species on the island, then subsequent hybridization may have bridged the genetic resources of these two closely related species, which are otherwise ecologically distinct, possibly helping them to respond more rapidly to new selection regimes in a changing climate.

In this study, we studied how the marginal populations of two closely related but ecologically distinct species (*R. palmatus* and *R. grayanus*) responded to postglacial climate change at the southern and northern range limits, respectively, and the genetic consequences. We hypothesized that differences in the species’ response to postglacial climate change led to range contacts, which may have resulted in increased opportunities for hybridization if the species were able to hybridize. To test this, we first examined the dynamics of the marginal populations of the species by coalescent analysis using nuclear sequence data. We then tested the hybridization of the contact zones along the altitudinal gradients on Yakushima Island at the species boundary by coalescent analysis using both nuclear and chloroplast sequences. Since we did not have enough paleobotanical information for the current species boundaries for *Rubus* or Rosaceae, we projected current and past distributions of the environmental envelop of these species, using an ensemble forecasting approach to compare the results of the genetic analysis.

## Results

### *Genetic diversity in* R. palmatus *and* R. grayanus

**Figure 1:**
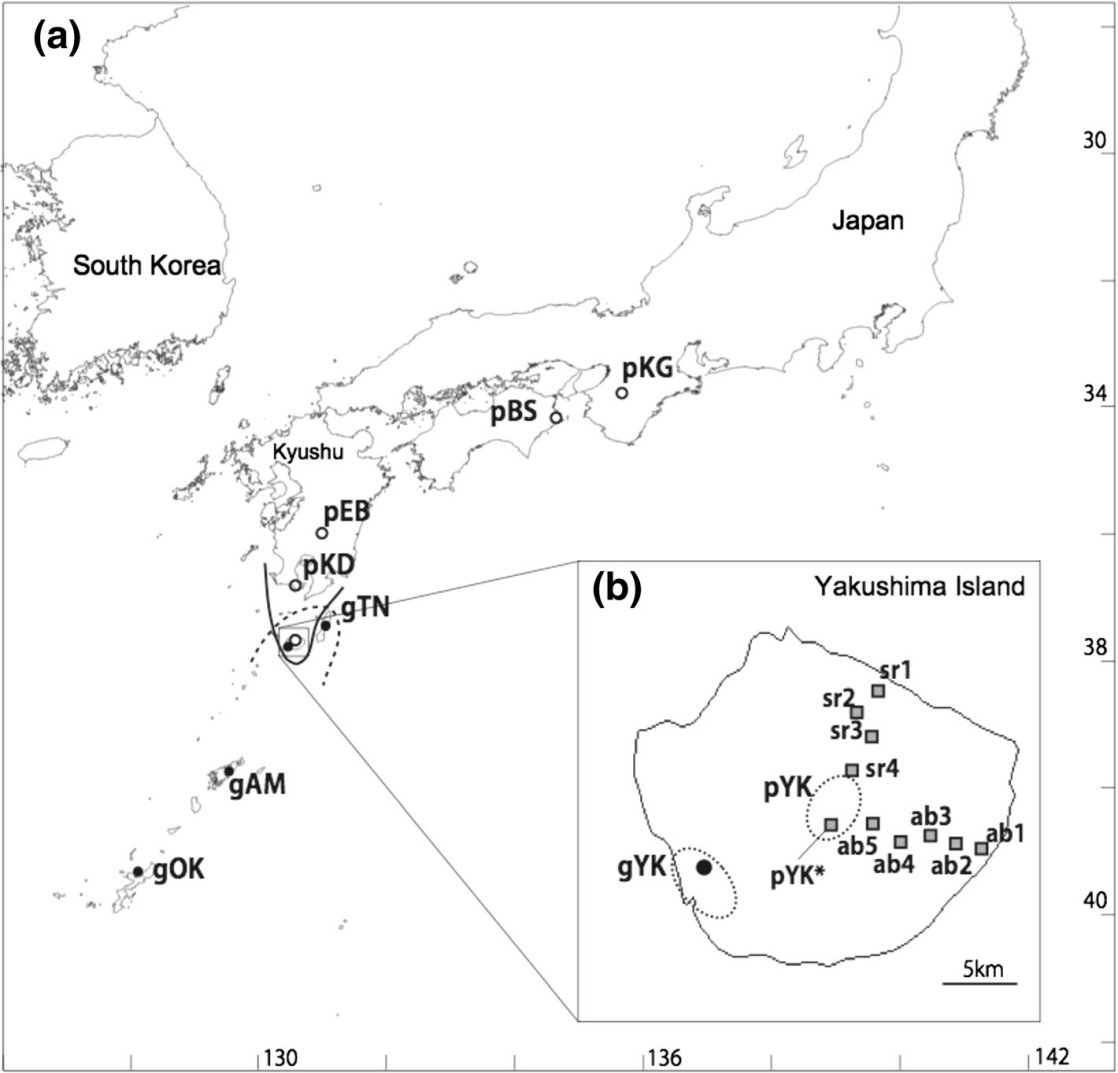
**Locations of sampled populations and species distributions. (a)** Open circles represent *Rubus palmatus* sample locations (pKG, pBS, pEB, pKD, and pYK), and closed circles indicate sample locations of *R. grayanus* (gOK, gAM, gYK, and gTN). *R. palmatus* southern limits (solid line) and *R. grayanus* northern limits (dashed line) are shown. Geographic coordinates and altitudes are given in Table 1. **(b)** Sampled populations along altitudes on Yakushima, where the two species’ distributions merged, are indicated as shaded squares (ab1, ab2, ab3, ab4, ab5, pYK* on Anbo Lane, and sr1, sr2, sr3, sr4 on Shiratani Lane). Some individuals from the pYK population (pYK*) were located along Anbo Lane at high altitude and included in the analysis of the contact zones. The dashed line indicates the approximate sampling areas for pYK and gYK populations.

The partial sequences of 12 functional nuclear loci, totaling 4,944 bp in size, were obtained from 54 and 56 individuals sampled from morphologically pure populations of *R. palmatus* and *R. grayanus*, respectively, around the species boundaries (Figure 1a, Table 1 and Additional file 1: Table S1; DDBJ:AB926443-AB927718; AB931175-AB932515; AB975184-AB975263). Sequence reads contained 106 bp of a non-coding region in the *COP1*-homolog gene and 92 bp in the *GSTF*-homolog gene. We identified 196 segregating sites when both species were considered, and 137 and 62 segregating sites within *R. palmatus* and *R. grayanus*, respectively. A total of 12 fixed sites between species in total were found in four loci (Additional file 1: Table S1). The eight other loci had only shared polymorphisms. The Wright fixation index, F_ST_, between the two species varied among loci, but was generally high (F_ST_ =0.371-0.859).

**Table 1:** **Sample population locations for the phylogeographic study at species boundaries and for the study at contact zones**

**Species**	**Location**	**Code**	**Species**	**Latitude**	**Lontitude**	**Altitude (m)**	**n**	**θ(total)**	**π(total)**
Species boundaries									
	Kongo	pKG	R. palmatus var. palmatus	34 25	135 40	1040	16	0.0029	0.0025
	Bisan	pBs	R. palmatus var. palmatus	34 04	134 32	240	12	0.0028	0.0031
	Ebino	pEB	R. palmatus var. palmatus	31 57	130 50	1195	30	0.0034	0.0038
	Kaimondake	pKD	R. palmatus var. palmatus	31 10	130 32	500	16	0.0013	0.0014
	Yakushima	pYK	R. palmatus var. yakumontanus	30 20	130 30	1100	32	0.0022	0.0019
	Yakushima	gYK	R grayanus	30 23	130 25	70	32	0.0018	0.0024
	Tanegashima	gTN	R grayanus	30 37	130 58	250	16	0.0013	0.0011
	Amami- Ōshima	gAM	R grayanus	28 25	129 30	100	32	0.0015	0.0017
	Okinawa	gOK	R.grayanus	26 34	128 00	160	32	0.0011	0.0007
Contact zones									
Anbo lane									
	Yakushima	ab1	-	30 306	130 643	45	16	0.0013	0.0011
	Yakushima	ab2	-	30 311	130 632	200	16	0.0018	0.0020
	Yakushima	ab3	-	30 316	130 623	400	16	0.0042	0.0039
	Yakushima	ab4	-	30 316	130 605	600	16	0.0037	0.0051
	Yakushima	ab5	-	30 317	130 586	900	16	0.0037	0.0032
	Yakushima	pYK*	-	30 20	130 30	1100	16	0.0011	0.0010
Shiratani lane									
	Yakushima	sr1	-	30 409	130 561	100	16	0.0012	0.0011
	Yakushima	sr2	-	30 394	130 569	400	16	0.0038	0.0035
	Yakushima	sr3	-	30.39	130 572	600	16	0.0042	0.0060
	Yakushima	sr4	-	30 376	130 573	760	16	0.0026	0.0023

The contact zone were located on the Yakushima where *R. palmatus*’ range and *R. grayanus*’ range are contacted. Some individuals in pYK, located on the Anbo Lane were also included in the analysis for contact zones (pYK*). n is the number of sequence data (2n: number of studied individuals); *θ*(total) and *π*(total) is gene diversity.

The average, within population nucleotide diversity, π (total), was generally higher in *R. palmatus* (π (total) = 0.0014-0.0038) than in *R. grayanus* (π (total) = 0.0007-0.0024; Table 1). Nucleotide diversity was highest in *R. palmatus* populations: pEB (0.0038) followed by pBS (0.0031). The highest nucleotide diversity in *R. grayanus* was found in gYK (0.0024) followed by gAM (0.0017) and the lowest was found at Okinawa (gOK, 0.0007). The levels of diversity were intermediate or high, within the range of silent nucleotide diversity in model and tree species [20].

Neutrality tests were carried out over loci; and the results for both Tajima’s D and Fu and Li’s D significantly departed from a neutral expectation in the Yakushima population (gYK) of *R. grayanus*. In *R. grayanus*, the southern populations (gOK) had significantly negative Tajima’s D (*α* = 0.05; Additional file 1: Table S2). The pEB population in *R. palmatus*, which had the highest nucleotide diversity, had a significantly positive Fu and Li’s D value. Fu and Li’s D was positive and marginally significant (*α* = 0.10) in the southernmost population of *R. palmatus* (pYK). None of the genes studied here were significant for the maximum likelihood analysis of the HKA test.

### Divergence and migration within species

**Figure 2:**
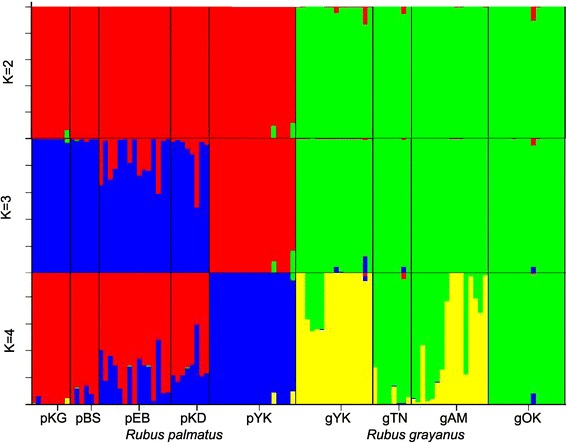
**Population structure of** ***Rubus palmatus*** **and** ***R. grayanus*** **based on SNPs from 12 nuclear genes.** The plots were obtained with the Bayesian clustering analysis program Structure [22].

As a result of the STRUCTURE analysis for the populations at the species boundaries, the most likely value of *K* was 2 when Evanno *et al.’s* [21] method was used, and 3 when Pritchard *et al.’s* [22] method was used (Figure 2).

The demographic parameters estimated in the IM models are listed in Table 2. Four additional models were run with different prior sets for a subset of models, and the demographic estimates were generally consistent regardless of priors (Additional file 1: Table S3). The posterior distribution of the divergence time in the two-population model with pYK and gYK (the tails of the distribution extend to zero) showed a clearer peak when the prior for migration rate was exponentially distributed, than when it was uniformly distributed (Additional file 1: Table S3). Thus, we used an exponential prior for the migration rates in further simulations (Table 2). Among *R. palmatus* populations, the lower 95% HPD for divergence time between each pair of populations was estimated to be before the LGM (18,000-20,000 Mya), except between the two eastern populations (pBS, pKG; 17,000 ya with 95% HPD 0–64,000 ya). Among *R. grayanus* populations, the more southerly populations (gOK, gAM) had deeper divergence compared to the more northerly ones. Estimates of divergence time between pairs of populations in *R. grayanus* were relatively smaller within the three northern populations (gYK, gTN, gAM) and the smallest divergence time was between the two northernmost populations, gYK and gTN (8,000 ya with 95% HPD 0–45,000 ya).

**Table 2:** **Estimated demographic parameters for the population pairs assuming isolation with migration**

	**Model**						
	**pop1, 2**	***t***	***θ***_***1***_	***θ***_***2***_	***θ***_***A***_	**2NM1 <2**	**2NM1>2**
Within *R. palmatus*							
	pYK, pKD	0.086 (0.025-0.318)	15.1 (2.6-16.9)	7.6 (2.6-16.9)	51.7 (22.9-98.5)	0.003 (0.0-0.010)	0.001 (0.0-0.006)
	pYK, pEB	0.110 (0.05-0.21)	17.5 (8.4-31.1)	27.5 (14.8-46.1)	59.8 (34.8-96.1)	0.000 (0.0-0.035)	0.000 (0.0-0.073)
	pEB, pKB	0.058 (0.016-0.145)	30.2 (13.6-58.3)	6.7 (2.1-16.8)	40.7 (22.8-68.0)	0.005 (0.0-0.022)	0.000 (0.0-0.006)
	pBS, pKG	0.017 (0.0-0.064)	8.1 (2.1-39.4)	28.8 (13.1-40.3)	22.8 (9.4-43.1)	0.000 (0.0-0.011)	0.000 (0.0-0.112)
	pEB, pKG	0.098 (0.042-0.189)	36.2 (20.0-62.4)	41.2 (19.1-86.4)	38.9 (20.9-63.8)	0.010 (0.0-0.024)	0.010 (0.0-0.032)
Within *R. grayanus*							
	gOK, gAM	0.038 (0.015-0.088)	9.9(3.2-24.9)	11.8 (4.9-25.7)	13.6 (5.9-27.2)	0.001 (0.0-0.100)	0.002 (0.0-0.010)
	gOK, gYK	0.040 (0.017-0.11)	7.3 (2.0-20.0)	10.8 (3.4-22.7)	18.4 (6.3-38.2)	0.001 (0.0-0.007)	0.001 (0.0-0.008)
	gOK, gTN	0.022 (0.005-0.062)	8.0 (1.6-30.1)	13.4 (2.0-134.3)	6.4 (1.8-14.9)	0.000 (0.0-0.011)	0.000 (0.0-0.043)
	gAM, gYK	0.015 (0.006-0.065)	4.8 (1.5-13.5)	9.8 (2.7-26.5)	22.3 (9.4-40.2)	0.000 (0.0-0.013)	0.000 (0.0-0.025)
	gAM, gTN	0.016 (0.005-0.061)	9.9 (1.9-32.1)	7.3 (0.5-56.3)	13.5 (4.5-26.6)	0.000 (0.0-0.011)	0.000 (0.0-0.017)
	gYK, gTN	0.008 (0.0-0.045)	5.5 (0.7-20.5)	2.2 (0.3-11.7)	13.6 (5.5-29.9)	0.000 (0.0-0.008)	0.000 (0.0-0.004)
Between species							
	pEB, gYK	0.870 (0.439-1.466)	37.9 (25.2-55.7)	17.0 (9.3-28.4)	24.8 (0.0-103.4)	0.009 (0.0-0.022)	0.007* (0.001-0.013)
	pKD, gYK	0.861 (0.425-1.470)	12.9 (6.1-23.4)	17.0 (9.3-27.9)	2.9 (0.0-133.8)	0.000 (0.0-0.008)	0.000 (0.0-0.010)
	pYK, gYK	1.07 (0.27-2.22)	17.5 (8.6-29.9)	16.9 (8.6-29.3)	0.9 (0 - 170.3)	0.022*** (0.005-0.057)	0.005** (0.0003-0.026)
	pEB, gTN	1.043 (0.549-1.625)	47.5 (32.0-67.0)	16.1 (7.9-27.9)	0.068 (0.0-112.0)	0.000 (0.0-0.026)	0.006** (0.001-0.012)
	pKD, gTN	0.961 (0.398-1.884)	16.6 (8.0-28.4)	17.0 (8.4-28.9)	47.0 (0.0-192.5)	0.000 (0.0-0.010)	0.000 (0.0-0.011)
	pYK, gTN	0.925 (0.389-4.54)	17.0 (9.3-27.9)	15.7 (7.5-26.6)	69.8 (0.0-355.2)	0.010*** (0.005-0.016)	0.005 (0.0-0.011)
	pEB, gAM	0.99 (0.44-1.67)	45.1 (29.0-66.4)	15.8 (7.7-27.9)	34.2 (0-123.1)	0.000 (0-0.041)	0.000 (0-0.019)

Highest probability densities (HPD) and 95% HPD in parentheses of demographic parameters, in isolation with migration models of two species populations (pop1 and pop2): divergence time in million years ago (*t*) of two focal populations, population size in thousands in population1 (θ_*1*_), population 2 (θ_*2*_), ancestral population (θ_*A*_), and migration rate from population 2 to 1 (2NM_1<2_) and from population 1 to 2 (2NM_1>2_).

Migration rates (2NM) were tested by likelihood ratio tests (Nielsen and Wakeley, 2001); *p < 0.05, **p < 0.01, ***p < 0.001.

The estimates of divergence time between the two species were approximately consistent among all pairs of populations (0.86–1.07 M years ago as HPD). A significant migration was detected only with the between-species models. These models detected significant migration from *R. palmatus* to *R. grayanus* (pEB to gYK, pYK to gYK, and pEB to gTN) and from *R. grayanus* to *R. palmatus* (gYK to pYK, and gTN to pEB).

### Genetic structure at the contact zones on Yakushima

**Figure 3:**
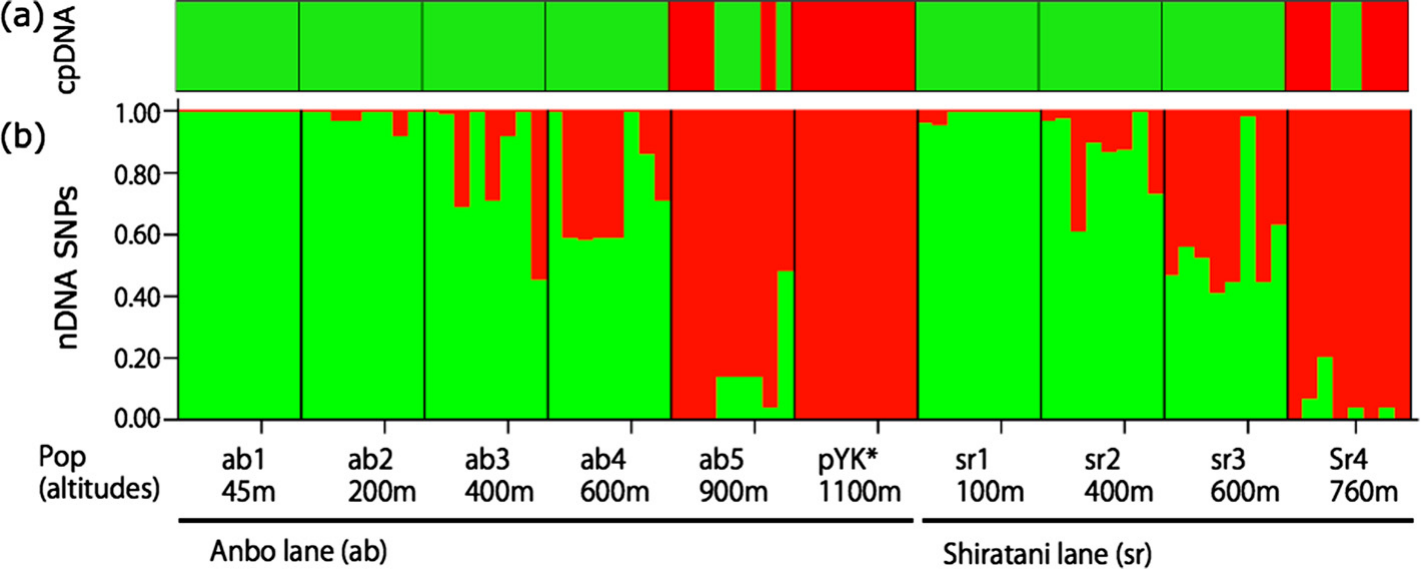
**Population structure of populations along altitude on Yakushima. (a)** Haplotypes of cpDNA (trnH-psbA intergenic region) illustrated in green (*R. grayanus*) and red (*R. palmatus*). **(b)** STRUCTURE analysis based on SNPs from 17 nuclear genes, assuming K = 2.

**Figure 4:**
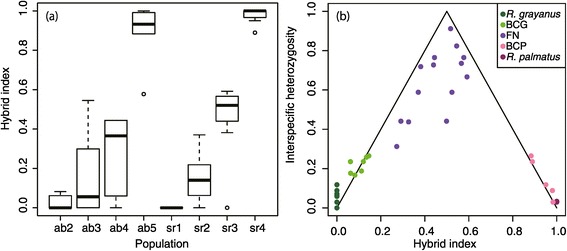
Hybrid index assuming that the pYK* (at the highest altitude) and ab1 (at the lowest altitude) populations are parental populations. (a) Hybrid index of each population, where hybrid index 0 = *R. grayanus* and 1 = *R. palmatus*. **(b)** Interspecific heterozygosity *vs* hybrid index based on SNPs of 17 nuclear genes. Colored dots indicate broadly defined hybrid classes [63]; BCG as backcrosses with *R. grayanus*; BCP as backcrosses with *R. palmatus*; FN as advanced-generation hybrids.

The partial sequences of 17 nuclear loci, in addition to one chloroplast locus, totaling 6302 bp in size, were obtained from 80 individuals sampled from the contact zones on Yakushima Island (Figure 1b, Table 1 and Additional file 1: Table S1). Most individuals from the contact zones had an *R. grayanus* cpDNA haplotype (Figure 3a). Assuming K = 2, a clinal genetic admixture was detected along the two altitudinal gradients on the island (Figure 3b). Clines were also observed in the hybrid index along the altitude (Figure 4a), assuming pYK* and ab1 populations as the parental populations based on the result of the STRUCTURE analysis. Among the 64 tested individuals, 32.8% were classified as pure *R. grayanus*, 15.6% as backcrosses with *R. grayanus*, 28.1% as advanced-generation hybrids, 12.5% as backcrosses with *R. palmatus,* and 10.9% as pure *R. palmatus* (Figure 4b). Interspecific heterozygosity ranged from 0 to 0.912, and some hybrids had lower interspecific heterozygosity than expected (Figure 4b). The IMa simulation, based on the putative parental populations (ab1, pYK*), estimated significant migration from *R. palmatus* to *R. grayanus* (LLR test; 4.633, p < 0.05), but gene flow in the opposite direction was not significant.

### Species distribution models

**Figure 5:**
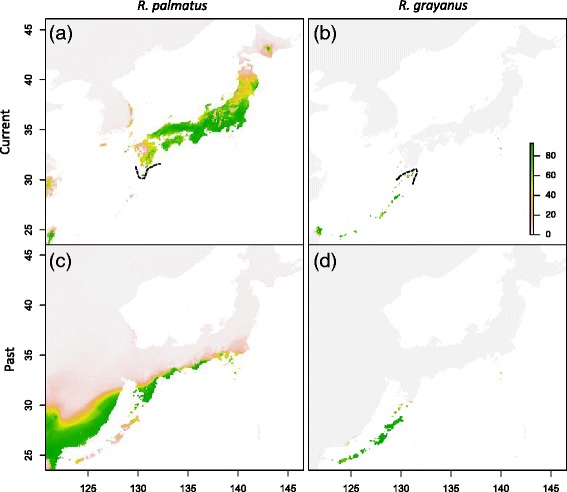
**An ensemble forecasting of current and past distribution of** ***R. palmatus*** **and** ***R. grayanus*** **based on the bioclimatic variables.** Sets of uncorrelated bioclimatic variables were used for each species. Probability of occurrence maps **(a, b)** and projected distribution of the suitable climates in the past (approx. 20,000 years ago; **c**, **d**) in *R. palmatus* **(a, c)** and *R. grayanus* **(b, d)**. Dashed lines indicate the known current southern and northern limits of *R. palmatus* **(a)** and *R. grayanus* **(b)**, respectively.

According to Thuiller *et al*. [23], the area under the curve (AUC) of an ROC for true skill statistics (TSS) and Kappa statistics indicated a good-to-excellent model performance, when averaged across all replications for all modeling techniques under current climatic conditions (AUC > 0.9, TSS > 0.8, and Kappa > 0.8; Additional file 1: Table S4); the expected Kappa value of CTA for *R. palmatus* = 0.74. The ensemble forecasting, based on six different modeling techniques, for the distributions of *R. palmatus* and *R. grayanus* under current climates predicted slightly wider distributions compared to the known distributions (dashed line indicates the known current southern and northern limits of *R. palmatus* and *R. grayanus* in Figure 5a and b).

Projections of past distribution during the LGM (~21,000 years BP) are shown in Figures 5c and d. The projection for *R. palmatus* shows a southern retreat from the major parts of its current distribution. The projection of past distributions of *R. grayanus* shows decreased probabilities of occurrence at the current northernmost range limits. Moreover, the most suitable habitats during the LGM were the southern islands, including Amami-Ōshima Island and Okinawa Main-Island (Figure 5d). A limitation in the accuracy may be due to the small amount of geo-reference data for *R. grayanus*, but in general, the current distribution had a higher occurrence probability in both species (see geo-reference location in Additional file 1: Figure S1).

## Discussion

### Range dynamics at species boundaries with postglacial climate change

The range shifts associated with climate change often leave a signature in the population genetic structure. Because of repeated bottlenecks during range expansion, the high-latitude limits of species that migrated after the LGM tend to have less genetic diversity than that of more southern locations [24,25], unless they are derived from an admixture of populations that originated from multiple refugia [26]. In contrast, the rear edge populations at low-latitudinal limits of species’ range can be slightly older because of latitudinal displacement of the distribution range, or can be more stable and the oldest populations within a species range, because of the persistence of relict populations during the climate changes. This has been observed in a number of species, based on phylogeographic surveys (reviewed in [6]). The range shift in response to climate change differs among populations and species. The differences might create opportunities to encounter other species, which may induce interspecific interactions such as competition and hybridization or lead to isolation from currently interacting species.

This comparative study of two related species, based on the coalescent analysis, suggests that the temperate species, *R. palmatus,* maintained its current southern limit populations on the island during the LGM and the population encountered expanding populations of its subtropical relatives, *R. grayanus,* after the LGM on the island. The relatively deep divergence without gene flow after divergence in populations within *R. palmatus* implies that the current populations were derived from multiple refugia. The lower bounds of the 95% HPD of the estimated time since divergence between the Yakushima population (pYK) and other nearby populations (pEB and pKD) are 25,000 years ago (86,000 and 111,000 years ago at HPD, respectively). Given that the LGM ended 18,000–20,000 years ago, Yakushima Island likely served as an isolated refugium for *R. palmatus* under the current climates and under past climates during the LGM. Yakushima Island was once geographically connected to the south of Kyushu Island, and probably served as a refugium for temperate evergreen forest species during the LGM [19,27]. In the case of *R. palmatus,* which is currently distributed at high altitude on the island, the island may have provided isolated habitats for the species, even during the LGM. The topography of the island may have offered a climatic niche for *R. palmatus* during the LGM and contributed to the stability of the southernmost population. Most of the species is endemic to this island, occurring at higher elevations [28], which may explain the historical isolation of the island in a phylogenetic context with cool temperate species. On the other hand, the island is less likely to have served as a refugium for the subtropical species, *R. grayanus*. Our analysis of *R. grayanus* suggests a recent range expansion from more southerly-located populations. We found a recent divergence among the northern populations of *R. grayanus*. The estimated divergence time of approximately 15,000 years ago, between the northern populations (gYK, gTN) and the nearest population (gAM), suggests a population decline or the complete retreat of the current northern tip of the distribution during the LGM. The ecological niche modeling also suggests both the stability of the southern population of *R. palmatus* and the population decline or complete retreat of *R. grayanus* from Yakushima Island during the LGM.

*R. grayanus* can be found on the strip of islands that extend to China, including Yakushima island, which indicates that the seeds have the capacity to disperse over sea, perhaps by animals [29,30] including migrant birds [31], as was observed in other *Rubus* species. Considering the high probability of a post-glacial population divergence on Yakushima and Tanegashima Islands from the more southerly located islands, the initial colonization may have occurred with seed dispersal by animals from nearby islands. After the divergences, the lack of any significant migration indicates that current seed dispersal between the islands is very rare. A rise in sea-level during the post-glaciation period increased the distances between islands, perhaps also decreasing the probability of seed dispersal among the islands.

We detected a significant gene flow between *R. palmatus* and northern *R. grayanus* populations. This violation of the IM model may have biased the parameter estimates, though the 95% HPD can often include the true values [32]. Nonetheless, *R. palmatus* likely kept its southern limits on the island and the range expansion of *R. grayanus* that was due to past climate change resulted in the current contact between the species on the island.

### Current and past introgression at species boundaries

Hybridization between *R. grayanus* and *R. palmatus* and asymmetric introgression with backcrosses were observed at intermediate altitudes on Yakushima Island, where the two species merged relatively recently at the species boundaries. This result agrees well with previous studies showing that the significant introgression was triggered by increased sympatry of species distributions following environmental changes [33,34], including contemporary climate changes [16]. In this study, we detected not only current active hybridization but also past introgression. The STRUCTURE analysis indicated a clear divergence between the morphologically pure species at the species boundary near Yakushima Island, suggesting that recent hybridization in these populations is limited. On the other hand, coalescence analysis that considers all migration since the two species diverged, indicated the presence of past hybridization events, probably as a consequence of past species contacts due to climate oscillations. The repeated range expansions and retreats [4], occurring with oscillations in climates, might have induced repeated contacts at the margins of the ranges for these two closely related, but ecologically distinct species, leaving a complex signature of introgression.

In the hybrid zones of *Rubus*, backcrosses with both parental species revealed clinal trends in the hybrid index. While hybridization and backcrosses could occur in both directions, most of the hybrids had *R. grayanus* as a seed donor and the introgression was asymmetric towards *R. grayanus* in the current hybrid zones on the island. Introgressive hybridization is occasionally asymmetric, which could be due to natural selection [12,13], pre- and post-zygotic reproductive isolation [35], or differences in population demography, such as relative abundance [36,37] and population growth rate resulting in “gene surfing” [38,39]. Relative abundance may play a role in asymmetric introgression since a rarer species would have a greater chance of receiving foreign genes from a more abundant species than vice versa, and first generations of hybrids tend to backcross with the most common parent at the local scale [36,37,40]. Based on our phylogeographic study, *R. grayanus* is the late colonizer on the island, perhaps starting from a small population size. Thus, the abundance theory does not fully explain the pattern. Instead, our observation is consistent with the expectation of gene surfing and introgression from an expanding species. Currat *et al*. [41] used simulations to predict that demographic difference on its own could cause asymmetric introgression from a local species to a recently colonized and expanding species until colonization is complete, because the number of copies of an allele introgressed in the incoming species will quickly increase due to its high rate of population growth. Thus, regardless of differences in abundance, asymmetric gene flow will take place from a local to a colonizing population. If the distribution of cpDNA reflects the population growth of *R. grayanus* as it expanded northwards during the past climate change, then *R. grayanus* may have more reproductive outputs compared to *R. palmatus*, which is supported by Currat *et al*.’s [41] prediction.

The observed structure of the hybrid zones was consistent with successive generations of hybrids. A range of interspecific heterozygosity in advanced-generation hybrids suggests that these populations corresponded to a mixture of early- and later-generation hybrids with recombination. We also found a discontinuity in the clinal hybrids zone (described in Figure 4), which suggests reproductive isolation between hybrids and one side of the parent species, *R. palmatus*. Steep clines have been observed with assortative mating and strong postzygotic barriers [42]. Since pre- and post-zygote barriers can result in asymmetric introgression [35], this may also be a cause of asymmetry of the hybrid zones. We are currently testing reproductive isolation by artificial crossing between *R. palmatus* and *R. grayanus*. Nonetheless, backcrossing with both ecologically divergent parental species was not a rare event in the hybrid zones, and it may lead to adaptive introgression by transferring adaptive traits at species boundaries with environmental selection [12,13,15]. Although some of our sequenced genes were found to be adaptive in other species; e.g., phytochrome in European aspen [43,44], we did not detect significant effects of selection in any of the loci we studied and these sequences behaved neutrally, at least in our dataset. Observations of genome-wide genetic variants and phenotypic variation in a common garden experiment of hybrids are currently being performed to detect natural selection for adaptive traits.

## Conclusions

In this study, we found significant differences in the response to past climate change in the populations of two ecologically distinct, but related species, at the margin of their species ranges. The fluctuations in range changes likely led to repeated contacts between closely related species thereby increasing the chance for hybridization. Genetic variation from other species can be a source of adaptive variation to respond to environmental changes as with standing variation and new mutation [45], and genes provided by introgression can be a crucial source of variation to respond to selection [46–49]. Introgressive hybridization induced by climate changes at the species boundaries, where populations may be isolated but still an important source for further range shift in response to climate change, could provide essential genetic variation to facilitate the recovery of population size and range expansion. Further detailed investigation of genomic clines and hybrid fitness along the hybrid zones at the margins of the ranges would provide insights into the roles of hybridization in population persistence and expansion in a changing environment.

## Methods

### Plant materials and sampling

Plant tissues of two *Rubus* species (diploid) were collected from 2010-2012 in Japan, including Yakushima Island: *Rubus palmatus* var*. yakumontanus* (pYK), *R. palmatus* var. *palmatus* (pEB, pKD, pBZ, pKG), and *R. grayanus* (gYK, gOK, gAM, gTN) (Table 1 and Figure 1a). In total, 54 and 56 individuals were collected from *R. palmatus* and *R. grayanus* populations, respectively. On Yakushima Island, the distribution of the two species is generally distinct: *R. palmatus* occurs in highlands (800 ~ 1200 m) and *R. grayanus* is found in lowlands (500 m at the highest). At intermediate altitudes; however, some individuals on a major logging lane (Anbo Lane), where narrow open habitats are available, are morphologically intermediate between *R. palmatus* and *R. grayanus* (Mishima *et al.,* unpublished data). Thus, we also collected tissue samples of 80 individuals along an altitudinal gradient on Yakushima Island (“contact zones”): 5 populations (ab1-5) and 4 populations (sr1-4) from two major logging lanes; Anbo and Shiratani Lanes, respectively. Some individuals of the pYK population were located at the highest altitude on Anbo Lane, and were included in the analysis as the pYK* population (Table 1 and Figure 1b). Although these two species are ecologically distinct, they are phylogenetically close. A phylogeny of 12 *Rubus* species distributed in Japan belonging to subgenus *Ideaobatus*, based on two regions (*rbcL* and *trnH-psbA*) of chloroplast DNA, indicate that *R. palmatus* and *R. grayanus* are sister species among the tested species (Mimura, unpublished data). All leaf samples were dried in silica gel before DNA extraction.

### Primer design and sequence data collection

Primers for nuclear genes were designed based on the *R. palmatus* EST libraries. The EST libraries were constructed from cDNA synthesized from *R. palmatus* mRNA samples from Yakushima Island. Sequences were generated using the GS FLX (454 Life Sciences, Branford, CT). The GS De Novo assembler version 2.3 (provided by 454 Life Sciences) was used for sequence assembly, and the quality trimming was performed with default settings. The function of homologous genes was predicted using *Arabidopsis* RNA protein reference sequences (http://blast.ncbi.nlm.nih.gov, threshold E-value 10^-20^ ). We found a homolog sequence as a hypothetical protein for some loci in the *Arabidopsis* reference sequences and in the reference sequences of other species (*Populus* and *Vitis*). Primers were designed based on the aligned sequences between *R. palmatus* and *R. grayanus* using Primer3 version 0.4.0 [50]. Twelve nuclear loci were sequenced for the populations at the species boundaries to infer past population dynamics of the two species. Since the contact zones on Yakushima Island are geographically limited, we sequenced 5 additional nuclear loci (giving a total of 17 nuclear loci) to increase the resolution of their genetic structure analysis, in addition to one chloroplast region (trnH-psbA) used to trace their maternal species. Loci information was described in Additional file 1: Table S1 in the Additional file of Supporting Information.

Genomic DNA was isolated from dried tissues using the modified CTAB method [51]. PCR reaction mixes contained 0.2 mM dNTPs, 1 × reaction buffer, 0.5 mM of each primer, 1 U of *Taq* DNA polymerase (TAKARA BIO) and approximately 20 ng of DNA. PCR amplifications were performed under the following conditions: 5 min at 94°C, followed by 30 cycles of 30 sec. at 94°C, 30 sec. at 55°C, and 1 min at 72°C. The amplified products were sequenced using ABI Prism BigDye Terminator v. 3.1 on an ABI3730 DNA Analyzer. Individual haplotypes were reconstructed using the PHASE algorithm [52].

### Summary statistics and population structure

We estimated the number of segregating sites, *S*, the total nucleotide diversity, and the nucleotide diversity within populations for the total (*π* (total), *θ* (total)), synonymous (*π* (s)), and non-synonymous (*π* (a)) sites [53]. The HKA test [54] was conducted to test for neutral evolution across loci: we used a maximum likelihood ratio test [55] to evaluate a model, assuming that one of the genes is not neutral, against a null model assuming that all genes are neutral. Statistical significance of Tajima’s D [56] and Fu and Li’s D (Fu and Li, [57]) was also tested with 1000 coalescent simulations using a standard neutral model. Simulations of neutrality tests were performed using the programs SITES and HKA (J. Hey https://bio.cst.temple.edu/~hey/software/software.htm).

The Bayesian clustering program STRUCTURE var. 2.3.3 [22] was used to assess the genetic clustering of individuals from the two species and the contact zones on Yakushima Island. We randomly collected one SNP from each locus. We performed five independent runs with a burn-in of 5.0 × 10^5^ and additional 5.0 × 10^5^ steps with the admixture model, and estimated log-likelihoods for each number of clusters (1 < *K* < 6) for the populations from the species’ ranges. For the contact zones on the island, we assumed *K* = 2, two gene pools from the two putative parental species.

### Coalescence analysis

Demographic parameters, including effective population size (*θ* for 4N_e_μ), migration rate (*m*), and divergence time (*t*) were estimated, assuming an Isolation-with-Migration (IM) model, using the IMa2 program [58,59]. The IMa2 program implements a Markov Chain Monte Carlo (MCMC) method to estimate posterior probability densities for the model parameters of the IM model. Since the model assumes no intralocus recombination, the minimum number of recombinations (*R*_*m*_) was estimated, and for each locus, we used a portion of the sequence where no recombination was found. We collected 5.0 × 10^5^ genealogies every 200 MCMC steps from three independent runs, each following a burn-in period of 5.0 × 10^5^ steps. The same uniform priors limits were set for the models within each species ([θ, t] = [5,10]) with a prior of migration rate per mutation event following an exponential distribution, with mean m* = 0.05. The infinite site model was applied to all loci, and 40 Markov-coupled chains were used. After failing to detect significant migration between populations within species in the two-population models, we ran models with a pair of allopatric and parapatric populations of both *R. palmatus* and *R. grayanus*. We limited our model to two populations, since we had sequence data from only 12 or 17 loci, and an insufficient amount of data can mislead the estimation of posterior probability density when more populations are considered [59]. For the contact zones, the coalescence analysis used only a model with pYK* and ab1, where the analysis of population structure suggested pure *R. palmatus* and *R. grayanus* populations, respectively, located at both edges of the contact zones. The divergence time was scaled using the geometric mean of the mutation rates per year per locus and assumed a generation time of 5 years. Since the mutation rate is not known in these *Rubus* species, we assumed those of two model species; *Arabidopsis thaliana* (1.5 × 10^-8^) [60] as the upper bound, and *Populus trichocarpa* (2.5 × 10^-9^) [61] as the lower bound. We used the mean substitution rates of the two model species for demographic parameters. The ratio of the average total genetic divergence to the average synonymous divergence (K (total)/Ks) was used to calculate the geometric mean of the substitution rate, using the synonymous substitution rate per site per year.

While our sampled populations violated some assumptions of the IM model [58], the violation of those assumptions, particularly population structure, had little effect on the estimates [32]. With limited data, priors may also have strong effects on posterior probability, especially for migration rates [59]. We tested different priors, and the posterior probabilities were reasonably robust (Additional file 1: Table S3). For instance, the posterior probabilities of divergence time between allopatric populations and between parapatric populations with significant migration rates were congruent.

### Hybrid parameters on Yakushima

The Hybrid index and interspecific heterozygosity were calculated using *introgress* [62]. They were based on randomly collected single SNPs from each of the 17 nuclear loci for individuals from the contact zones on Yakushima Island. Based on the STRUCTURE analysis of the contact zones, we defined pYK* and ab1 populations as parental *R. palmatus* and *R. grayanus* populations, respectively. We broadly defined the hybrid classes, as suggested by Hamilton *et al.* [63] (i.e. backcrosses to either parent when hybrid index is more than 85% but not 100%; advanced-generation hybrids (FN) when hybrid index is more than 15% and less than 85%). We used this broad classification of hybrids [63], because it requires fewer assumptions than the other Bayesian approach, i.e., New Hybrids [64].

### Ecological niche modeling

#### Occurrence and environmental data

Herbarium occurrence data was collected from the Global Biodiversity Information Facility (http://www.gbif.org/). Limited data is available for *R. grayanus* occurrence and we added our sampling and observation locations as occurrence points. In total, 436 geo-reference data points were made for *R. palmatus* and 13 were made for *R. grayanus*. Current and past (at LGM) climate variable data was obtained from WorldClim version 1.4 [65]. We used the data layer based on simulations that used the Community Climate System Model (CCSM). The resolutions of current climate and past layers were 30 arcsec and 2.5 arcmin, respectively. Among the 19 available climate variables, those which are not highly correlated (Pearson correlation coefficient r < |0.7|) were used in the model to avoid multicollinearity problems, leaving sets of 6 climate variables for *R. palmatus* and *R. grayanus*: annual mean temperature, mean diurnal range, mean temperature of wettest quarter, annual precipitation, precipitation seasonality, precipitation of coldest quarter for *R. palmatus,* and annual mean temperature, isothermality, max temperature of warmest month, annual precipitation, precipitation of driest month, precipitation seasonality for *R. grayanus*.

### Ensemble modeling

A number of species distribution models (SDM) are available, making the selection of the most appropriate methodology for each case study a challenge [66]. To account for inter-model variability, we used an ensemble forecasting approach based on six commonly used models in the bioclimatic niche modeling package BIOMOD2 [67,68] in R (R Development Core Team, [69]). The models included generalized linear models (GLM), generalized additive models (GAM), multivariate adaptive regression spline (MARS), classification tree analysis (CTA), random forest (RF), and the maximum entropy approach (MAXENT). Most of the modeling techniques used in BIOMOD2 require absence data, but the best strategy for the pseudo-absence selection depends on the models. Established selection guidelines are not yet available for an ensemble model of multiple models [70]. We generated 5 different sets of randomly chosen 500 pseudo-absences. The models were calibrated for each pseudo-absence dataset (the absences were weighted equally to the presences) with 10-fold cross-validation using a 70% random sample of the observed data, and model performance was assessed using the remaining 30% of the data. MAXENT [71] projects the distribution with presence-only data, compared to random background pixels. We included a result of MAXENT with a maximum of 10,000 background points and cross-validation with 12 replicates in Additional file 1: Figure S2.

### Availability of supporting data

The datasets supporting the results of this article are included with the article and additional files. The sequence datasets supporting the results of this article are available in the DNA Data Bank of Japan (DDBJ: http://www.ddbj.nig.ac.jp/).

## Additional file

Additional file 1: Table S1. Loci used in this study. **Table S2.** Summary statistics and neutrality tests of populations at species boundaries. **Table S3.** Other priors tested in IMa2. **Table S4.** Model evaluation for ecological niche modeling. **Figure S1.** Locations of occurrence data used for ecological niche modeling. **Figure S2.** Projection of current and past distribution of *R. palmatus* and *R. grayanus* using the Maxent.
