# Supplementary material for: Range shift and introgression of the rear and leading populations in two ecologically distinct Rubus species
Source: BMC Evol Biol. 2014 Oct 25;14:209. doi: 10.1186/s12862-014-0209-9 (PMC4221717; doi:10.1186/s12862-014-0209-9)
Supplement: Additional file 1: Table S1. — Loci used in this study. Table S2. Summary statistics and neutrality tests of populations at species boundaries. Table S3. Other priors tested in IMa2. Table S4. Model evaluation for ecological niche modeling. Figure S1. Locations of occurrence data used for ecological niche modeling. Figure S2. Projection of current and past distribution of R. palmatus and R. grayanus using the Maxent. [file 12862_2014_209_MOESM1_ESM.docx]

Additional file

Tables

Table S1. Loci used in this study

Table S2. Summary statistics and neutrality tests of populations at species boundaries

Table S3. Other priors tested in IMa2

Table S4. Model evaluation for ecological niche modeling

Figures

Figure S1. Locations of occurrence data used for ecological niche modeling

Figure S2. Projection of current and past distribution of *R. palmatus* and *R. grayanus* using the Maxent

Tables

Table S1. Loci used in this study

Loci used and summary statistics at species boundaries and contact zone (Table 1). The putative gene functions are annotated based on the database of *Arabidopsis thaliana* reference proteins (min. threshold e-value was less than 10^-20^). bp: total length of sequence used for analysis; n(total) the total number of phased sequences; S: the total number of segregating sites; fixed S: the number of segregating sites between species that are fixed within a species; F_ST_ : population differentiation. *COP1-* and *GSTF-*homolog gene sequences included non-coding regions. Asterisk indicates chloroplast DNA region.

Table S2. Summary statistics and neutrality tests of populations at species boundaries

Population summary statistics for the populations at the species boundaries (Table 1). The parameters shown are the number of haplotype studied: n; the average number of segregating sites: S; nucleotide diversity (θ and π) for total, synonymous (s), non-synonymous (a), and non-cording. Tajima’s D and Fu & Li D were also calculated and simulated. Mean values and lower and higher 95% intervals were simulated with 1000 coalescent simulations and compared with the observed value to test its significance. ^+^p<0.10, *p < 0.05, **p < 0.01.

Table S3. Other priors tested in IMa2

Other priors tested in this study and highest probability density (HPD) with 95% HPD in parentheses, in isolation with migration models of two species populations (pop1 and pop2): divergence time in million years ago (*t*) of two focal populations, population size in thousands in population1 (θ*_1_*), population 2 (θ*_2_*), ancestral population (θ*_A_*), and migration rate from population 2 to 1 (2NM_1<2_) and from population 1 to 2 (2NM_1>2_). Migration rates (2NM) were tested by likelihood ratio tests (Nielsen and Wakeley, 2001); *p < 0.05, **p < 0.01, ***p < 0.001. We used two migration priors for independent runs; a uniform distribution on [0,1] for migration rate, and an exponential distribution with mean m* = 0.05.

Table S4. Model evaluation for ecological niche modeling

Averaged TSS, AUC and Kappa values for model evaluation. The values were averaged from 10 runs of each modeling technique for each of 5 sets of pseudo-absence datasets.

Figures


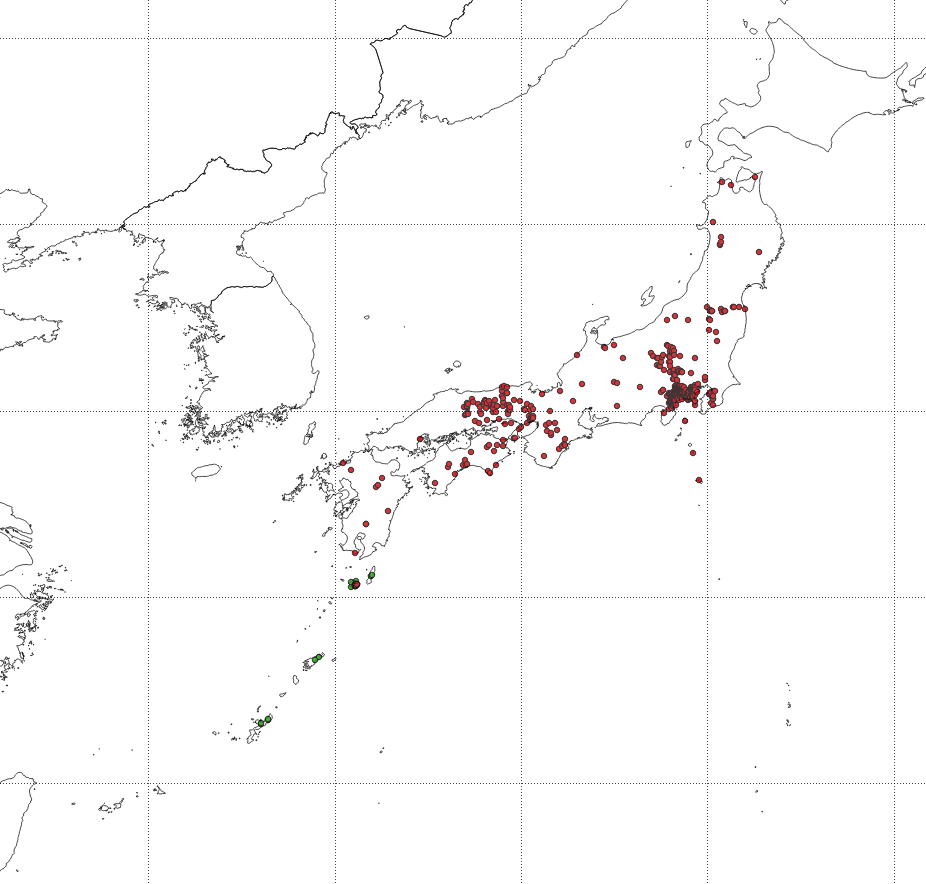


Figure S1. Locations of occurrence data used for ecological niche modeling. Red and green circles represent occurrence data for *R. palmatus* and *R. grayanus,* respectively. Occurrence data were obtained from GBIF (<http://www.gbif.org>) and also included the sampling sites for phylogeographic analysis in this study.

Figure S2. Projection of current (a, b) and past (c, d) distribution of *R. palmatus* and *R. grayanus* using the Maxent. Maxent is a modeling technique that use only presence data. A dozen of runs were cross-validated with random seeds and averaged for each species projection.
